# Effect of acidosis in the late-finishing phase on rumen fermentation in feedlot steers

**DOI:** 10.1093/tas/txae084

**Published:** 2024-05-27

**Authors:** Haley F Linder, Larry L Berger, Joshua C McCann

**Affiliations:** Department of Animal Sciences, University of Illinois Urbana-Champaign, Urbana, IL 61801, USA; Department of Animal Sciences, University of Illinois Urbana-Champaign, Urbana, IL 61801, USA; Department of Animal Sciences, University of Illinois Urbana-Champaign, Urbana, IL 61801, USA

**Keywords:** acidosis, feedlot, late-finishing phase

## Abstract

The objective was to determine the effects of induced acidosis in the late-finishing phase on rumen fermentation in feedlot steers. Eleven ruminally cannulated steers (body weight [BW] = 795 kg ± 54) were blocked into two groups based on initial BW. For 195 d prior to the start of the study, cattle were consuming a basal finishing diet (60% dry-rolled corn, 15% modified distillers grains, 15% corn silage, and 10% ground corn-based supplement). Steers were randomly assigned to one of the two treatments: control (**CON**), or induced acidosis (**ACD**). Both treatments were fasted for 24 h then fed the basal finishing diet. Steers on the ACD treatment received 0.05% of BW of wheat starch via rumen cannula at 0800 and 2000 hours on day 1 and ad libitum refeeding following the fast. On days 1 and 2, CON steers were provided 25% of allotted feed every 6 h. Rumen fluid was collected every 4 h during the challenge period (hours 0 to 48), and 0, 6, and 12 h after feeding during the recovery period (hours 54 to 96). Rumen fluid was analyzed for pH, ammonia, volatile fatty acids (**VFA**), and lactate. Fecal grab samples were collected every 8 h to determine fecal pH. A treatment × day interaction (*P *= 0.03) was observed for dry matter intake during the challenge period with steers on the ACD treatments consuming more on day 1 than CON steers. Intake was not different on day 2 (*P* = 0.88). A treatment × hour effect (*P *< 0.01) was observed for ruminal pH during the challenge period with the ACD steers having a lesser pH than CON from hours 12 to 32. Duration of time below a pH of 5.6 during the challenge period was greater (*P* < 0.01) for ACD steers than CON. During the challenge period, a treatment × time interaction (*P *= 0.04) was observed for total VFA concentration with ACD steers having greater total VFA concentration from hours 12 to 36. Acetate to propionate ratio (A:P) was affected by treatment × hour (*P* = 0.04) with CON steers having greater A:P from hours 28 to 48. Rumen ammonia and lactate concentrations did not differ (*P* ≥ 0.25) between treatments or the interaction with time. Challenge and recovery period fecal pH were not affected (*P *≥ 0.13) by treatment, time, or their interaction. Recovery period ruminal pH was not different (*P *= 0.99) between treatments. For the recovery period, total VFA and ammonia concentration were not affected by treatment, time, or their interaction (*P *≥ 0.07). Ruminal pH and VFA were affected in the initial 48 h of induced acidosis in the late-finishing phase.

## Teaser

A bout of ruminal acidosis was induced in steers adapted to a high-concentrate diet to understand its effects on the rumen fermentation parameters of steers during the late-finishing phase.

## Introduction

In the finishing phase of beef cattle production, high-grain diets are fed to cattle to meet increased energy requirements for rapid weight gain. However, greater dietary inclusion of rapidly fermentable carbohydrates increases the rate and amount of volatile fatty acids (VFA) produced by microbial fermentation, resulting in a decrease in rumen pH. Ruminal acidosis occurs when ruminal pH values drop below 5.6 (Nagaraja and Titgemeyer, 2007). This disorder is costly to the beef industry as it negatively impacts animal health and performance (Stock, 2000). Cattle that experience ruminal acidosis are at risk of reduced feed intake (Britton and Stock, 1989), bloat (Cheng et al., 1998), laminitis (Nocek, 1997), and rumenitis (Glock and DeGroot, 1998). Additionally, repeated bouts of ruminal acidosis damage rumen epithelial cells, hindering absorption and barrier function (Steele et al., 2011). Disruptions in rumen epithelial barrier function allow bacteria and toxins to translocate from the rumen to the liver via the portal vein, resulting in abscess formation (Nagaraja and Chengappa, 1998). Lesions in the ruminal wall have been positively associated with liver abscesses (Rezac et al., 2014). Cattle that spend more time at ruminal pH < 5.8 are at greater risk of developing severe liver abscesses (Wiese et al., 2017).

The transition from a forage-based to concentrated-based diet is considered the period with greatest risk for ruminal acidosis (Bevans et al., 2005; Brown et al., 2006). However, feedlot cattle can still have an acidotic ruminal pH for several hours a day even after adapting to high-concentrate diets (Wierenga et al., 2010). Ruminal acidosis prevalence and severity increase with days on feed (Castillo-Lopez et al., 2014; Cusack et al., 2021). Carcass weights and the duration of time in which feedlot cattle receive a concentrate-based diet have increased over the last fifteen years (Peel, 2023). Thus, the acidosis risk in the late-finishing phase may continue to increase as cattle continue to spend more days on feed.

Developing a greater understanding of this disorder throughout the feedlot phase will allow for continued advancement in nutritional management of feedlot cattle. In the current literature, few studies have investigated ruminal acidosis in cattle at the end of the finishing phase. Therefore, the objective was to determine the effects of induced acidosis in the late-finishing phase on rumen fermentation in feedlot steers.

## Materials and Methods

The experimental protocol was approved by the Institutional Animal Care and Use Committee at the University of Illinois at Urbana Champaign (Protocol # 22161). Eleven ruminally cannulated Angus and Simmental × Angus steers (body weight [**BW**] = 795 ± 54 kg) were utilized in two blocks based on initial BW, with six and five animals in each block, respectively. Although 12 steers were initially ruminally cannulated, one steer was removed due to mortality unrelated to treatment. Prior to the start of the experiment, animals were consuming a finishing diet (Table 1) for 195 to 210 d.

**Table 1.** Diet composition and chemical analysis

Ingredient	% Dry matter
Dry-rolled corn	60
Modified wet distiller grains	15
Corn silage	15
Supplement	
Ground corn	7.62
Limestone	1.59
Urea	0.60
Trace mineral premix^*^	0.10
Rumensin 90	0.02
Tylosin 40	0.01
Choice white grease	0.07
Chemical analysis	
Dry matter	59.4
Organic matter	97.5
Crude protein	12.9
Neutral detergent fiber	18.6
Acid detergent fiber	7.9
Ether extract	

^*^8.5% Ca, 5% Mg, 7.6% K, 6.7% Cl, 10% S, 0.5% Cu, 2% Fe, 3% Mn, 3% Zn, 278 mg/kg Co, 250 mg/kg I, 150 Se, 2,205 KIU/kg Vit A, 662.5 KIU/kg Vit D, and 22,047.5 IU/kg Vit E.

For the experiment, steers were housed in a climate-controlled metabolism barn set to 18.3 °C, at the University of Illinois Beef Cattle and Sheep Field Research Laboratory in Urbana, IL. Individual tie stalls (2.3 × 1.3 m) had a feed bunk and non-siphoning, automatic water bowl. The experimental period was a total of 12 d. Day −7 through −1 was the adaptation period. On day 0, all steers were fasted from feed with full access to water for 24 h. At the conclusion of the fast, steers received the same basal finishing diet. Sample collection occurred on days 1 to 4. For sample collection days, the challenge period was defined as hours 0 to 48 and the recovery period as hours 54 to 96. Times were relative to the conclusion of the fast (0800 hours on day 1).

Within each weight block, cattle were stratified based on average rumen pH recorded 6 h after feeding on two consecutive days during the adaptation period. After the stratification, one of the two treatments were then randomly assigned: control (**CON**; *n* = 5), or induced acidosis (**ACD;***n* = 6). Acidosis steers received 0.05% of BW of wheat starch via rumen cannula at 080 and 2000 hours on day 1. Following the fast, ACD steers were refed ad libitum. To reduce animal-to-animal variation in acidosis susceptibility (Mohammed et al., 2012), ACD cattle needed to consume a minimum of 1.9% of their BW in feed dry matter (DM) by h 12 on d 1. Any remaining feed of that allotment was given via the rumen cannula at hours 12. Control steers did not receive a dose of ruminal wheat starch. Control steers were also fasted to mimic the physiological conditions resulting from the fast. However, since CON steers received the basal finishing diet after the 24-h fast, their rate of feed consumption was reduced to minimize pH depression. Steers on CON were offered 0.6% of BW in feed DM every 6 h on days 1 and 2. Additionally, the feed was removed if rumen pH dropped below 5.5 and replaced once pH returned above 5.5.

### Sample Collection

BWs were collected prior to feeding on the day of steers entering the metabolism barn. Feed samples were collected on days 1 to 4 and all feed refusals were weighed back daily and approximately 200 g subsampled. Feed and feed refusal samples were stored at −20 °C for later analysis. During the challenge period, rumen fluid was collected from the ventral sac via an indwelling suction strainer every 2 h to measure rumen pH with a benchtop pH meter (Accumet Basic AB15; Fisher Scientific, Hampton, NH). Every 4th h, a larger volume of rumen fluid, approximately 50 mL, was collected. A 2 mL aliquot of fluid was added to a microcentrifuge tube for lactate analysis. For volatile fatty acid (**VFA**) analysis, 4 mL of rumen fluid was added to a tube containing 4 mL of 2N HCl. For ammonia analysis, 8 mL of rumen fluid was added to a tube containing 2 mL of 2N HCl. All tubes were immediately frozen and stored at −20 °C until laboratory analysis. Every 8 h, a fecal grab sample was collected. Ten grams were subsampled and mixed with 10 mL of distilled water to measure fecal pH. During the recovery period, rumen fluid was sampled using the method previously described at hours 6 and 12 after feeding for rumen pH, VFA, and ammonia, concentration. Fecal pH was taken at hour 12 after feeding. All samples were collected using the same methods as previously described.

### Laboratory Analysis

Feed samples were composited within the block while ort samples were composited by animals. Feed, fecal, and orts composites were dried in 55 °C oven and ground through a Wiley mill (1-mm screen, Arthur H. Thomas, Philadelphia, PA, USA). Composites were analyzed for DM (24 h at 105 °C), neutral detergent fiber and acid detergent fiber (using Ankom Technology method 5 and 6, respectively; Ankom^200^ Fiber Analyzer, Ankom Technology, Macedon, NY, USA), nitrogen by combustion to estimate crude protein (Leco TruMac; LECO Corporation, St. Joseph, MI, USA), ether extract (Ankom method 2; Ankom Technology), and organic matter (600 °C for 12 h; Thermolyte muffle oven Model F30420C; Thermo Scientific, Waltham, MA, USA).

Rumen fluid samples were thawed and analyzed for ammonia concentrations with the method described by Broderick and Kang (1980) using a spectrophotometer (SpectraMax QuickDrop; Molecular Devices, San Jose, CA, USA). Concentrations of VFA were determined by gas chromatography (HP5890 series, Hewlett-Packard, Wilmington, DE, USA) on a glass column using an adapted method from Erwin et al. (1961). Total lactate concentrations were analyzed via HPLC (Agilent 1,260 Infinity, Agilent, Santa Clara, CA, USA). A Supelcogel C610H ion-exclusion column (Sigma-Aldrich, Inc, St. Louis, MO, USA) was used, and separation was carried out utilizing 5 mM sulfuric acid. The flow rate of the eluent was 0.5 mL/min, with an injection volume of 10 µL. Column temperature was 70 °C and run time was 30 min. Based on ruminal pH values, only time points hours 0 to 36 were included in lactate analysis.

### Statistical Analysis

#### Data were analyzed as a randomized block design with steer as the experimental unit and block as a random effect.

The MIXED procedure of SAS 9.4 (SAS Inst. Inc., Cary, NC) was utilized for all variables except lactate concentration, with the fixed effect of treatment. Repeated measures were used for rumen pH, fecal pH, ammonia, and VFA with fixed effects of treatment, time, and the interaction of treatment and time. Covariance structure was selected based on fit statistics. Compound symmetry was used for challenge period ruminal pH and NH_3_, and first-order autoregressive was selected for challenge period VFA and fecal pH. For the recovery period, first-order autoregressive was used as the covariance structure for all variables. Nadir pH was defined as the lowest rumen pH value for each steer during either the challenge or recovery data set. Duration of time spent under benchmark 5.6 was calculated by taking the sum of the time points steers were at or below a rumen pH of 5.6 during the challenge period and multiplying by 120 to determine minutes. DM intake (**DMI**) and duration of time spent under benchmark pH were analyzed with the fixed effect of treatment, day, and their interaction. Lactate concentration data were analyzed using the GLIMMIX procedure of SAS 9.4 (SAS Inst. Inc., Cary, NC) using a gamma distribution to account for non-normality, with the fixed effect of treatment, and the random effect of block, and steer nested within treatment. Least squared means were back-transformed and estimated using the ILINK statement. Significance was declared at *P* ≤ 0.05 and tendencies were discussed at 0.05 < *P* ≤ 0.10.

## Results

**Figure 1.**
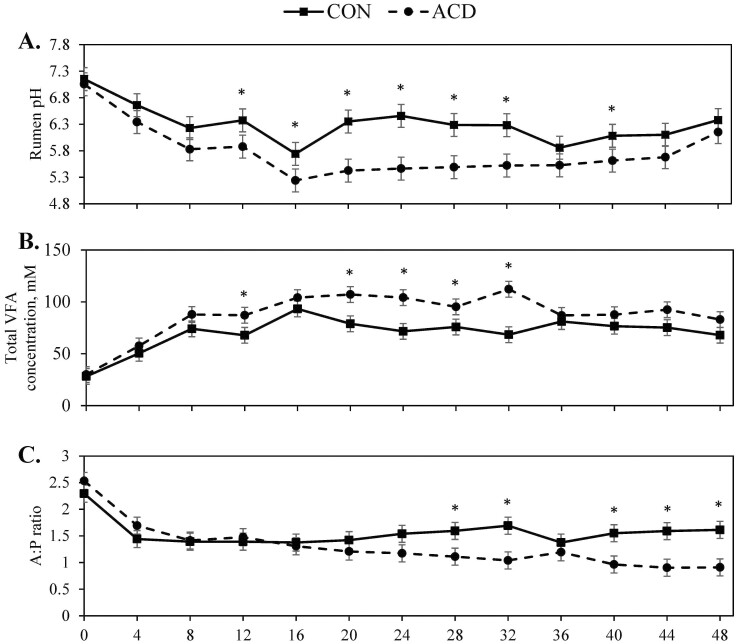
Effect of induced acidosis in the late-finishing phase on rumen pH (A), total volatile fatty acids concentration (B), and acetate:propionate (A:P) ratio (C). Treatments consisted of CON (control) or ACD (induced acidosis). X-axis refers to the hour of the experiment. All variables were affected by treatment × time (*P* < 0.01). Pairwise treatment differences were sliced by time and indicated by * when *P* ≤ 0.05.

A treatment × day interaction (*P *= 0.03) was observed for DMI during the challenge period (Table 2). Steers on the ACD treatment consumed 5.6 kg more on day 1 than CON steers by design, but intake was not different from CON steers on day 2 (Table 2). During the recovery period, intake was not affected by treatment, day, or their interaction (*P *≥ 0.17). For the challenge period, a treatment × hour (*P* = 0.01; Figure 1) effect was observed for ruminal pH with the ACD steers having a lesser pH than CON from hours 12 to 32. Nadir pH was affected by treatment (*P *< 0.01) with ACD steers having a lesser nadir pH than CON steers, 5.09 and 5.44, respectively (Table 3). Duration of time spent under a pH of 5.8 and 5.6 was only affected by treatment (*P *< 0.01). Control steers spent 250 and 170 min/d below 5.8 and 5.6, respectively. In contrast, steers on the ACD treatment spent 907 and 787 min/d below 5.8 and 5.6, respectively.

**Table 2.** Effect of induced acidosis in the late-finishing phase on dry matter intake (DMI) of steers

	Treatment					*P*-value^*^		
Item	CON		ACD		SEM	Trt	Day	Trt × day
Challenge period	day 1	day 2	day 1	day 2				
DMI, kg	10.13^a^	7.80^a^	15.72^b^	8.04^a^	1.613	0.02	<0.01	0.03
Recovery period	day 3	day 4	day 3	day 4				
DMI, kg	12.17	10.70	11.17	12.07	1.231	0.82	0.74	0.17

^a,b^Within a row, common superscripts indicate no significant difference between means, *P* > 0.05.

^*^Trt = treatment effect; Trt × day = treatment × day effect.

CON, control; ACD, induced acidosis.

**Table 3.** Effect of induced acidosis in the late-finishing phase on ruminal pH

	Treatment			*P*-value^*^		
Item	CON	ACD	SEM	Trt	Day	Trt × day
Challenge period nadir pH^†^	5.44	5.09	0.168	0.07	—	—
Recovery period nadir pH^‡^	5.41	5.28	0.132	0.38	—	—
Time < 5.8 min	250^a^	907^b^	173.1	<0.01	0.32	0.42^3^
Time < 5.6 min	170^a^	787^b^	160.5	<0.01	0.44	0.34^3^

^a,b^Within a row, common superscripts indicate no significant difference between means, *P* > 0.05.

^*^Trt = treatment effect; Trt × day = treatment × day effect.

^†^Challenge period = hours 0 to 48 relative to conclusion of fast.

^‡^Recovery period = hours 54 to 96 relative to conclusion of fast.

CON, control; ACD, induced acidosis.

During the recovery period, there was a tendency for a treatment × time interaction for rumen pH (*P *= 0.08), as ACD steers only had a lesser pH than CON steers at hour 84 (Table 4). Recovery nadir pH was not different between ACD and CON steers (*P* = 0.37; Table 3). Fecal pH in the challenge period and recovery was not affected (*P* ≥ 0.18) by treatment, time, or their interaction (Table 5).

**Table 4.** Effect of induced acidosis in the late-finishing phase on recovery period ruminal pH

	Treatment^1^			*P*-value^2^		
Item	CON	ACD	SEM	Trt	Time	Trt × time
Ruminal pH			0.257	0.99	<0.01	0.08
54 h	5.79	5.80				
60 h	5.76	5.98				
72 h	6.08	6.39				
78 h	5.92	5.91				
84 h	5.74	5.29				
96 h	5.85	5.77				

^*^Trt = treatment effect; Trt × time = treatment × time effect.

CON, control; ACD, induced acidosis.

**Table 5.** Effect of acidosis in the late-finishing phase on fecal pH

	Treatment^1^			*P*-value^*^		
Item	CON	ACD	SEM	Trt	Time	Trt × time
Challenge fecal pH^†^			0.257	0.18	0.41	0.58
8 h	6.54	6.45				
16 h	6.57	6.48				
24 h	6.93	6.38				
32 h	6.39	6.48				
40 h	6.40	6.00				
48 h	6.71	6.45				
Recovery fecal pH^‡^			0.217	0.74	0.77	0.70
60 h	6.56	6.44				
84 h	6.45	6.45				

^*^Trt = treatment effect; Trt × time = treatment × time effect.

^†^Challenge period = hours 0 to 48 relative to conclusion of fast.

^‡^Recovery period = hours 54 to 96 relative to conclusion of fast.

CON, control; ACD, induced acidosis.

**Figure 2.**
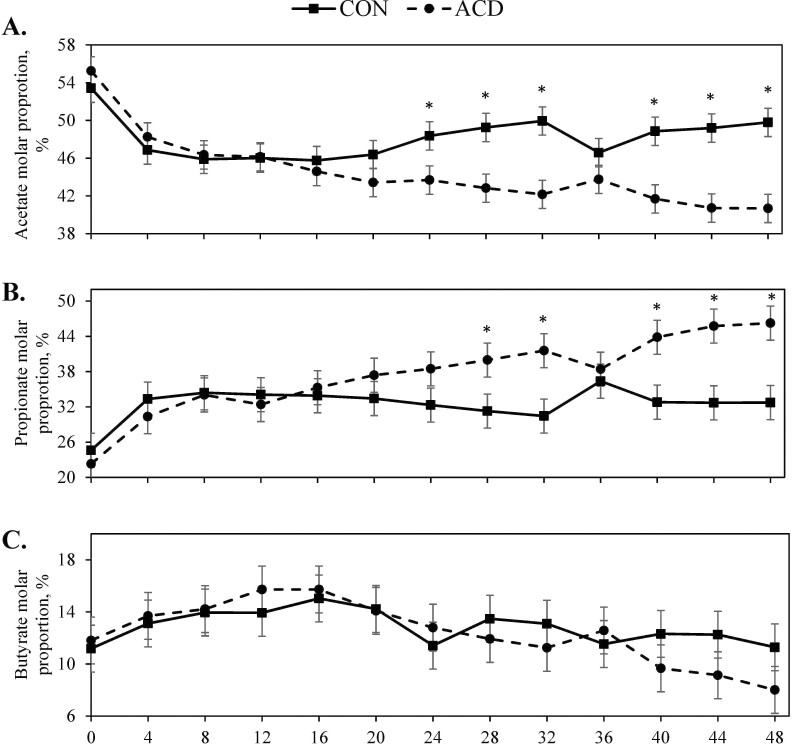
Effect of induced acidosis in the late-finishing phase on acetate molar proportion (A), propionate molar proportion (B), and butyrate molar proportion (C). Treatments consisted of CON (control) or ACD (induced acidosis). X-axis refers to the hour of the experiment. Acetate and propionate were affected by treatment × time (*P *< 0.01). Butyrate tended to be affected by hour (*P *= 0.09). For treatment × time interactions, pairwise treatment differences were sliced by time and indicated by * when *P* ≤ 0.05.

A tendency for a treatment × time interaction (*P* = 0.07) was observed for total VFA concentration during the challenge period with ACD steers having greater total VFA concentration from hours 12 to 32 (Figure 1). A treatment × hour interaction (*P *< 0.01) was observed for acetate molar proportion with CON having a greater percentage acetate at hours 24 through 32 (Figure 2). Additionally, there was a treatment × hour interaction (*P* < 0.01) for propionate molar proportion with ACD steers having greater propionate at hours 28 and 32, and then at hours 40 and 44. Butyrate was not affected (*P* ≥ 0.45) by treatment × hour or treatment. Acetate to propionate ratio (A:P) was affected by treatment × hour (*P* = 0.04) with CON steers having greater A:P after hour 28 (Figure 1). Isobutyrate molar proportion was affected (*P *< 0.01) by treatment and time (Table S1). Isovalerate molar proportion was affected (*P *≤ 0.04) by treatment and time. For both isobuyrate and isovalerate, molar proportions were greatest at hour 0 and then decreased over the 48 h challenge period. Steers on the CON treatment had greater isobutyrate and isovalerate molar proportion than ACD steers. Rumen ammonia and lactate concentrations did not differ (*P* ≥ 0.25) by treatment or the interaction with time (Table 6). Both ruminal ammonia and lactate concentration were greatest at h 0 (*P* ≤ 0.03).

**Table 6.** Effect of induced acidosis in the late-finishing phase on challenge period ruminal ammonia and lactate concentration

	Treatment			*P*-value^*^		
Item	CON	ACD	SEM^†^	Trt	Time	Trt × time
Ammonia, mM			8.584	0.63	<0.01	0.96
0 h	14.34	14.36				
4 h	1.59	2.41				
8 h	3.87	4.05				
12 h	1.83	1.58				
16 h	3.19	3.94				
20 h	3.83	3.97				
24 h	4.08	3.95				
28 h	4.41	4.31				
32 h	7.31	5.87				
36 h	9.19	5.13				
40 h	5.80	5.04				
44 h	6.12	4.71				
48 h	5.20	4.01				
Lactate, mM			—	0.26	0.03	0.37
0 h	3.80	3.95				
4 h	0.22	4.26				
8 h	1.79	1.74				
12 h	0.22	0.22				
16 h	0.41	0.36				
20 h	1.23	0.22				
24 h	0.22	0.50				
28 h	0.22	0.60				
32 h	0.22	1.20				
36 h	0.22	0.32				

^*^Trt = treatment effect; Trt × time = treatment × time effect.

^†^SEM not included for lactate due to transformed data.

CON, control; ACD, induced acidosis.

For the recovery period, total VFA concentration was not affected by treatment, time, or their interaction (*P *≥ 0.13; Table 7). There was a tendency for a treatment × time interaction (*P *= 0.10) for acetate molar proportion with CON steers having a greater molar proportion at hours 54 and 60. Propionate molar proportion was affected by treatment × time (*P *< 0.01) with ACD steers having greater propionate molar percentage only at hour 54. A treatment × time interaction (*P *< 0.01) was also observed for butyrate molar proportion as ACD had greater butyrate molar percentage at hours 54 and 60. There was only a tendency for a time effect (*P *= 0.06) observed for ammonia.

**Table 7.** Effect of induced acidosis in the late-finishing phase on recovery period rumen fermentation parameters

	Treatment			*P*-value^*^		
Item	CON	ACD	SEM	Trt	Time	Trt × time
Ammonia, mM			1.615	0.38	0.06	0.14
54 h	4.91	5.11				
60 h	4.07	5.95				
72 h	4.66	7.71				
78 h	6.52	9.42				
84 h	7.19	5.74				
96 h	6.14	4.84				
Total VFA, mM			10.743	0.23	0.26	0.13
54 h	90.77	81.65				
60 h	90.27	74.24				
72 h	79.95	66.90				
78 h	87.49	81.74				
84 h	80.01	97.19				
96 h	89.28	78.18				
VFA, % total mM						
Acetate			2.868	0.69	0.72	0.10
54 h	46.95	42.47				
60 h	45.01	41.68				
72 h	42.49	44.43				
78 h	41.91	44.66				
84 h	42.12	42.89				
96 h	44.88	42.21				
Propionate^†^			4.370	0.72	0.71	<0.01
54 h	34.84	43.56				
60 h	37.42	43.66				
72 h	41.59	40.11				
78 h	42.85	38.89				
84 h	42.62	40.36				
96 h	39.96	41.00				
Butyrate			2.399	0.39	0.39	<0.01
54 h	13.01^a^	7.29^b^				
60 h	12.63^a^	7.11^b^				
72 h	11.11	10.04				
78 h	10.80	11.46				
84 h	10.80	12.09				
96 h	10.59	10.16				

^a,b^Within a row, common superscripts indicate no significant difference between means, *P* > 0.05.

^*^Trt = treatment effect; Trt × time = treatment × time effect.

^†^Least square means were sliced by time, but no treatment effects (*P* < 0.05) were observed.

CON, control; ACD, induced acidosis.

## Discussion

Feedlot cattle are at an increased risk of developing ruminal acidosis due to dietary inclusions of rapidly fermentable carbohydrates. Although acidosis prevalence has not been reported in production feedlot cattle, acidotic conditions in the rumen (pH < 5.6) are likely common based on typical finishing diets (Samuelson et al., 2016). In this study, ruminal pH decreased from 0 to 16 h for both treatments. However, ACD steers experienced a greater depression in ruminal pH over time and a slower rate of recovery during the challenge period. Given the steers on both treatments were fasted and received a concentrate-based diet at the conclusion of the fast, the depression in ruminal pH during the first 16 h of the experiment for both treatments is indicative of an increased rate of microbial fermentation. Restricted refeeding rate of CON steers also contributed to their lesser VFA concentrations. The amount and rate of feed consumption both impact the rate at which VFA are produced and subsequently the decrease in rumen pH (Allen, 1997). Furthermore, the ruminal dose of wheat starch at hours 0 and 12 for ACD steers likely also contributed to the greater depression in pH. This inclusion provided additional nonstructural carbohydrates from a different feed source. Wheat has a faster rate of starch digestion than corn and would be fermented more rapidly (Stock, 2000). By increasing ruminal VFA concentration through substrate amount and availability, ruminal pH decreased at a greater rate following the induction of the acidosis challenge for the ACD steers than CON.

Nadir pH values (5.09 and 5.44) would both be classified as subacute acidosis for both ACD and CON steers. However, the duration of which ruminal pH remains below 5.6 is also crucial in classifying acidosis (Owens et al., 1998). The ACD treatment steers in this study had an average duration of ruminal pH < 5.6 for 787 min/d on d 1 and 2. Similarly, Bevans et al. (2005) reported beef heifers undergoing a subacute acidosis challenge spent approximately 660 min/d below a pH of 5.6. However, the cattle utilized by Bevans et al. (2005) had not received a grain-based diet prior to the start of the experiment. There was no difference in time spent < 5.5 during an acidosis challenge between heifers that received a 90% concentrate diet for either 34 or 8 d (Schwaiger et al., 2013). Thus, even with increased days on feed and dietary adaptation to finishing diets, cattle in the late-term finishing phase are still susceptible to extended amounts of time at a pH below 5.6.

Control steers also experienced a period of time < 5.6 (170 min/d). Beef steers with typical feed consumption patterns spent 195 min/d at a pH below 5.5 during the last 50 d of consuming a barley-based diet (Castillo-Lopez et al., 2014). Dairy cattle fed > 60% concentrate diets spent between 121 and 219 min/d below a pH of 5.6 (Gozho et al., 2005, 2006). Even in dairy cows receiving a 50% concentrate diet, average duration at a pH < 5.6 was 118 minutes (Khafipour et al., 2009). In the aforementioned studies, prior to the start of the experiment, cattle received a diet containing 50% concentrate. Thus, the length of time CON steers were below a pH of 5.6 aligns with prior studies feeding high-grain diets to cattle and supports the altered feed intake pattern used for CON steers.

The duration of time an animal spends below acidotic thresholds of rumen pH values can increase their risk of rumen epithelium damage and liver abscesses. During the last 5 wk of the finishing period, Wiese et al. (2017) reported steers with liver abscesses or rumen scarring spent more time below a ruminal pH of 5.8 compared with steers without pathology (547 vs. 365 min, respectively). However, there was no difference in the minimum and mean ruminal pH values between steers with or without liver and rumen pathology (Wiese et al., 2017). While pH was recorded using an indwelling device over several weeks, steers without pathology spent more time below 5.8 than the CON steers in the current study. Considering the ACD steers spent 904 min/d below 5.8 ruminal pH during the challenge, they would be at risk for developing rumen epithelial damage or liver abscesses.

In the current study, manipulation of feed intake patterns allowed for both the induction and control of ruminal acidosis. A 24 h-fast ensured cattle would rapidly consume a large amount of the finishing diet, resulting in an increased amount of VFA production. While there were differences in DMI between ACD and CON steers, these differences were accounted for by the fact that CON steer’s intake rate was limited. For reference, during the adaptation period, steers consumed approximately 1.7% of BW on a DM basis. In the first 12 h, all CON steers consumed their allotted amount of feed (1.25% BW on a DM basis) compared with ACD steers consuming 1.9% BW on a DM basis. By modifying the rate of feed consumption for CON steers, the amount of substrate available for microbial fermentation was controlled. When the rate of production of VFA exceeds the rate of absorption or outflow from the rumen, VFA accumulation decreases ruminal pH (Gäbel et al., 1991). Thus, by slowing the rate of feed intake, VFA production rate did not exceed absorption rate to the same degree in CON steers as in ACD steers. However, the impact of the rate of intake on pH differences cannot be separated from the consumption of starch in this study. During the recovery period, feed intake was not different between the treatments, as feed intake was not manipulated or controlled.

The differences in ruminal pH and feed intake support the differences observed in total VFA concentration between the ACD and CON steers. Total VFA concentration peaked at 112 mM in ACD steers compared with 81 mM in CON steers. While other studies of SARA in beef cattle have reported VFA concentrations greater than 120 mM in experimentally induced subacute acidosis, greater grain inclusion in these diets (>85%) may account for the greater VFA concentration compared with the current study (Goad et al., 1998; Bevans et al., 2005; Schwaiger et al., 2013). Despite receiving the same diet as CON steers, ACD steers had increased propionate molar proportion but decreased acetate molar proportion during the challenge period. However, ruminal dosing of wheat starch at h 0 and 12 not only provided an additional form of substrate for ACD steers, but also one that yields greater propionate production (Ørskov, 1986). Isovalerate and isobutyrate molar proportions were greatest at h 0 for both treatments, which may been a consequence of the fast. Similarly, plasma isovalerate and isobutyrate concentrations were increased in steers following a fast (Blum et al., 1981). Branched-chain amino acids are precursors for these VFA, which can be derived from feed protein or utilization of dead bacterial cells (Miura et al., 1983). Branched-chain VFA are essential to celluloytic bacteria growth, and these bacteria mainly are responsible for their synthesis (Andries et al., 1987). Thus, continued fermentation of slowly degradable fiber by celluloytic bacteria during fasting could shift the rumen proportion of VFA toward isovalerate or isobutyrate.

Lactate accumulation characterizes acute acidosis compared with subacute acidosis (Nagaraja and Titgemeyer, 2007). Ruminal lactate concentration exceeds 50 mmol/L in acute acidosis, but is below 5 mmol/L in subacute acidosis (Nagaraja and Titgemeyer, 2007). Other studies which induced SARA in beef cattle reported lactate concentrations less than 5 mmol/L (Brown et al., 2000; Bevans et al., 2005; Blanch et al., 2009). Similarly, lactate concentration was less than 4 mmol/L in the current study. Preventing lactate accumulation helps to maintain ruminal pH above acute acidotic levels as it is a stronger acid than VFA (Goad et al., 1998). Lactate is an intermediate product of ruminal fermentation and is still produced during non-acidotic conditions. However, bacteria that can metabolize lactate into propionate can prevent its accumulation and subsequent decline in rumen pH (Mackie and Gilchrist, 1979). These bacterial species’ growth rates are inhibited at a pH below 5.0 prompting lactate accumulation during acute acidosis (Therion et al., 1982). The nadir value for ruminal pH for ACD steers in this study was 5.09, which is above the threshold pH for negatively affecting lactate-utilizing bacteria. Additionally, risk of rumen lactate accumulation may decrease with days on feed. Lactate-utilizing capacity of the rumen increases with adaptation to a finishing diet and increased ruminal lactate concentration (Kunkle et al., 1976). Cattle that had received a 90% concentrate diet for 34 d did not have lactate accumulation during an acidosis challenge (Schwaiger et al., 2013). Therefore, the absence of ruminal lactate accumulation in the current study could contribute to an increase in lactate-utilizing capacity from the length of time on a finishing diet. Alterations in the rumen microbial populations due to the fast may be responsible for the greater lactate concentrations at hour 0 for both treatment groups. Fermentable nonstructural carbohydrates in the rumen likely decreased during the fast. This may have resulted in less substrate for lactate-utilizing bacteria, which could slow their growth rates (Counotte and Prins, 1981), and may have allowed for a small amount of lactate accumulation in the fasted rumen.

Fecal pH was not affected by treatment in our study during the challenge or recovery period despite treatment effects on ruminal pH and DMI. In subacute acidosis, given the more chronic and less severe ruminal pH depression compared with acute acidosis, fecal pH may be less likely to be impacted. During SARA, fecal pH depression is not as severe as what occurs ruminally (Plaizier et al., 2022). Thus, given the extent and severity of the acidosis bout in this experiment, it would have been unlikely to have observed an effect on fecal pH. For steers in the late-finishing fecal pH would not be a helpful indicator to identify animals with this level of acidosis.

Further research into acidosis in late-finishing beef cattle is warranted as days on feed continue to increase in the cattle feeding industry. While there is literature regarding acidosis in cattle adapted to grain feeding, there is no data on cattle that have been consuming a finishing diet for > 150 d. While this study aimed to understand the effects of induced acidosis on rumen fermentation, acidosis susceptibility was not evaluated. Understanding factors, such as intake variation or duration of time on feed, that contribute to acidosis risk will improve the management of finishing beef cattle. Additionally, the prevalence of acidosis in late-finishing cattle is unknown and requires future investigation.

## Conclusion

Ruminal acidosis was successfully induced in feedlot steers in the late-finishing phase. Reducing the rate of feed intake of CON steers minimized the duration of time that ruminal pH was below 5.6, even with steers consuming a grain-based diet following a fast. Volatile fatty acid concentration was increased in ACD steers, but there were no differences in lactate concentration between treatments. Fecal pH did not differ between treatments and should not be considered an indicator of acidosis in late-finishing cattle. Induced acidosis in late-finishing steers affected VFA production and pH during the initial 48 h of the bout. With increasing days on feed during the finishing phase, a greater understanding of ruminal function and acidosis is critical for improved animal productivity and health.

## Supplementary Material

txae084_suppl_Supplementary_Materials
