# Supplementary material for: Effect of acidosis in the late-finishing phase on rumen fermentation in feedlot steers
Source: Transl Anim Sci. 2024 May 27;8:txae084. doi: 10.1093/tas/txae084 (PMC11143494; doi:10.1093/tas/txae084)
Supplement: txae084_suppl_Supplementary_Materials [file txae084_suppl_supplementary_materials.docx]

Supplemental material

**Effect of acidosis in the late-finishing phase on rumen fermentation in feedlot steers**

Haley F. Linder^*^, Larry L. Berger^*^, Joshua C. McCann^*1^

^*^ Department of Animal Sciences, University of Illinois Urbana-Champaign, Urbana, IL, USA, 61801

^1^ Corresponding author: jcmccan2@illinois.edu

| **Table S1.** Effect of induced acidosis in the late-finishing phase on challenge period branch chain VFA concentration | | | | | | |
| --- | --- | --- | --- | --- | --- | --- |
|  | Treatment^1^ | |  | *P*-value^2^ | | |
| Item | CON | ACD | SEM | Trt | Time | Trt × time |
| VFA, % total mM |  |  |  |  |  |  |
| Valerate |  |  | 0.249 | 0.28 | 0.74 | 0.18 |
| 0 h | 1.68 | 1.53 |  |  |  |  |
| 4 h | 1.53 | 1.40 |  |  |  |  |
| 8 h | 1.58 | 1.38 |  |  |  |  |
| 12 h | 1.36 | 1.40 |  |  |  |  |
| 16 h | 1.60 | 1.37 |  |  |  |  |
| 20 h | 1.39 | 1.69 |  |  |  |  |
| 24 h | 1.64 | 1.92 |  |  |  |  |
| 28 h | 1.40 | 2.12 |  |  |  |  |
| 32 h | 1.57 | 2.22 |  |  |  |  |
| 36 h | 2.01 | 1.87 |  |  |  |  |
| 40 h | 1.66 | 2.17 |  |  |  |  |
| 44 h | 1.61 | 1.99 |  |  |  |  |
| 48 h | 1.57 | 1.98 |  |  |  |  |
| Isobutyrate |  |  | 0.366 | <0.01 | <0.01 | 0.91 |
| 0 h | 2.91 | 2.86 |  |  |  |  |
| 4 h | 1.41 | 1.54 |  |  |  |  |
| 8 h | 0.91 | 0.68 |  |  |  |  |
| 12 h | 1.11 | 0.75 |  |  |  |  |
| 16 h | 0.67 | 0.44 |  |  |  |  |
| 20 h | 1.06 | 0.52 |  |  |  |  |
| 24 h | 1.12 | 0.45 |  |  |  |  |
| 28 h | 1.14 | 0.48 |  |  |  |  |
| 32 h | 1.08 | 0.43 |  |  |  |  |
| 36 h | 0.76 | 0.56 |  |  |  |  |
| 40 h | 0.92 | 0.45 |  |  |  |  |
| 44 h | 0.92 | 0.45 |  |  |  |  |
| 48 h | 1.13 | 0.63 |  |  |  |  |
| Isovalerate |  |  | 0.852 | 0.04 | <0.01 | 0.16 |
| 0 h | 6.21 | 6.18 |  |  |  |  |
| 4 h | 3.77 | 4.60 |  |  |  |  |
| 8 h | 3.27 | 3.27 |  |  |  |  |
| 12 h | 3.51 | 3.51 |  |  |  |  |
| 16 h | 3.04 | 2.52 |  |  |  |  |
| 20 h | 3.54 | 2.81 |  |  |  |  |
| 24 h | 5.17 | 2.60 |  |  |  |  |
| 28 h | 3.47 | 2.61 |  |  |  |  |
| 32 h | 3.89 | 2.39 |  |  |  |  |
| 36 h | 2.75 | 2.74 |  |  |  |  |
| 40 h | 3.46 | 2.09 |  |  |  |  |
| 44 h | 3.36 | 1.87 |  |  |  |  |
| 48 h | 3.41 | 2.24 |  |  |  |  |
| ^1^ CON = control; ACD = induced acidosis.  ^2^ Trt = treatment effect; Trt × time = treatment × time effect. | | | | | | |

| **Table S2.** Effect of induced acidosis in the late-finishing phase on recovery period branch chain VFA concentration | | | | | | |
| --- | --- | --- | --- | --- | --- | --- |
|  | Treatment^1^ | |  | *P*-value^2^ | | |
| Item | CON | ACD | SEM | Trt | Time | Trt × time |
| VFA, % total mM |  |  |  |  |  |  |
| Valerate |  |  | 0.447 | 0.20 | 0.10 | 0.20 |
| 54 h | 1.75 | 2.54 |  |  |  |  |
| 60 h | 1.82 | 3.27 |  |  |  |  |
| 72 h | 1.74 | 1.76 |  |  |  |  |
| 78 h | 1.73 | 1.89 |  |  |  |  |
| 84 h | 1.86 | 2.08 |  |  |  |  |
| 96 h | 1.67 | 2.01 |  |  |  |  |
| Isobutyrate |  |  | 0.819 | 0.05 | 0.34 | 0.32 |
| 54 h | 0.59 | 0.71 |  |  |  |  |
| 60 h | 0.54 | 2.89 |  |  |  |  |
| 72 h | 0.76 | 1.08 |  |  |  |  |
| 78 h | 0.66 | 0.79 |  |  |  |  |
| 84 h | 0.59 | 0.53 |  |  |  |  |
| 96 h | 0.70 | 2.53 |  |  |  |  |
| Isovalerate |  |  | 0.267 | 0.52 | 0.07 | <0.01 |
| 54 h | 2.87^a^ | 1.83^b^ |  |  |  |  |
| 60 h | 2.59^a^ | 1.81^b^ |  |  |  |  |
| 72 h | 2.31 | 2.67 |  |  |  |  |
| 78 h | 2.05 | 2.39 |  |  |  |  |
| 84 h | 2.03 | 2.14 |  |  |  |  |
| 96 h | 2.20 | 2.19 |  |  |  |  |
| ^a,b^ Within a row, common superscripts indicate no significant difference between means, *P* >0.05  ^1^ CON = control; ACD = induced acidosis.  ^2^ Trt = treatment effect; Trt × time = treatment × time effect. | | | | | | |
